# Complete chloroplast genome sequence of *Punica granatum* ‘Nana’ (Lythraceae) and phylogenetic analysis

**DOI:** 10.1080/23802359.2020.1764401

**Published:** 2020-05-14

**Authors:** Jie Wang, Zhi-Qiang Wu, Li Ma, Cui-Hua Gu

**Affiliations:** a School of Landscape and Architecture, Zhejiang A & F University, Hangzhou, China; b Zhejiang Provincial Key Laboratory of Germplasm Innovation and Utilization for Garden Plants, Zhejiang A & F University, Hangzhou, China; c Key Laboratory of National Forestry and Grassland Administration on Germplasm Innovation and Utilization for Southern Garden Plants, Zhejiang A & F University, Hangzhou, China; d Shenzhen Branch, Guangdong Laboratory for Lingnan Modern Agriculture, Genome Analysis Laboratory of the Ministry of Agriculture, Agricultural Genomics Institute at Shenzhen, Chinese Academy of Agricultural Sciences, Shenzhen, China

**Keywords:** *Punica granatum* ‘Nana’, chloroplast genome, Lythraceae, phylogenetic analysis

## Abstract

Pomegranate (*Punica granatum* L.) is of great significance both as a fruit tree and an ornamental plant. Hereon, we sequenced and characterized the complete chloroplast genome of *Punica granatum* ‘Nana’ and performed phylogenetic analysis concerning related species. It turned out that the length of chloroplast genome sequence reached 158,639 bp and exhibited a four-conjoined structure, i.e., a large single copy region (LSC, 89,022 bp), a small single copy region (SSC, 18,685 bp) and twain inverted repeat regions (IRa and IRb, 25,466 bp). 112 unique genes were identified, consisting of 78 protein-coding genes, four ribosomal RNA (rRNA) genes and 30 transfer RNA (tRNA) genes. The result of phylogenetic analysis based on Neighbor-joining (NJ) method was consistent with that of Bayesian inference (BI), which strongly supported that *Punica granatum* ‘Nana’ was close to its original species *Punica granatum* and they together had a close relationship with *Heimia myrtifolia* within Lythraceae.

Pomegranate (*Punica granatum* L.), belonging to Lythraceae, is not only economically important in agricultural production as a welcome fruit tree, but also an excellent ornamental plant in horticulture for its showy flowers (Qin and Shirley 2007). It originates from central Asia and nowadays has been cultivated in tropical and subtropical regions worldwide(Yan et al. 2019). The long history of planting pomegranate has witnessed many varieties and cultivars with good taste and abundant progenies (Al Khayri et al. 2019). Hence, we described the traits of *Punica granatum* ‘Nana’ chloroplast genome and performed phylogenetic analysis, which would promote genetic and breeding research within this genus.

Fresh leaves of *Punica granatum* ‘Nana’ were collected in the nursery of Zhejiang A&F University, Hangzhou, Zhejiang province, China (30°13′48″N, 119°43′12″E). The specimen was stored at Herbarium of Zhejiang A & F University (specimen code ZAFU1912245). Extracting total genomic DNA was done according to the method proposed by Doyle (1987) and Yang et al. (2014). Following the establishment of a sequencing library based on purified DNA, paired-end reads were primarily obtained utilizing the Illumina High-throughput Sequencing technology, and then Trimmomatic v0.3(Bolger et al. 2014) worked to filter raw reads. The process of de novo assembly was accomplished by CLC v9.11 (Nicolas et al. 2017). The alignment of contigs was under the BLAST algorithm (Johnson et al. 2008) with *Punica granatum* plastid genome as reference (Gu et al. 2019). The genome was annotated using DOGMA v1.2 (Wyman et al. 2004) and submitted to GenBank (Accession number: MN833212). MsatCommander v0.8.2.0 (Faircloth 2008) was utilized to identify simple sequence repeats (SSRs). Plastid circular map was drawn via OGDRAW v1.3.1 illustrated by Greiner et al. (2019).

The chloroplast genome size of *Punica granatum* ‘Nana’ reached 158,639 bp, exhibiting a typical four-conjoined structure, i.e., a large single copy region (LSC, 89,022 bp), a small single copy region (SSC, 18,685 bp) and twain inverted repeat regions (IRa and IRb, 25,466 bp). The overall GC content was 36.92%, and detailedly, corresponding contents to LSC, SSC and IR regions were 34.89%, 30.63% and 42.78%, respectively. 53 simple sequence repeats (SSRs) were found out, among which 50(94.34%) were mono-repeats, 1(1.89%) were di-repeats and 2(3.77%) were tri-repeats. A total of 112 unique genes were detected, including 78 protein-coding, four ribosomal RNA (rRNA) genes and 30 transfer RNA (tRNA). There were 83 genes found in LSC, with 12 in SSC and 17 in IR regions, respectively. Eight protein-coding and six tRNA genes contained only one intron whereas three genes (*ycf*3, *clp*P and *rps*12) contained two. Gene *rps*12, particularly, was trans-spliced, characterized by first exon locating in LSC and the other two in IR regions.

To further explore the phylogeny of *Punica granatum* ‘Nana’, we obtained chloroplast genome sequences of 14 related species within Lythraceae from GenBank and selected two species of Onagraceae as outgroups. Sequence alignment was implemented via MAFFT v7 (https://mafft.cbrc.jp/alignment/server/index.html) (Katoh et al. 2019), and then jModelTest v2.1.7 (Darriba et al. 2012) was utilized to decide the optimal model. Afterward, we used MrBayes v3.2.6 (Ronquist et al. 2012) and Mega vX (Kumar et al. 2018), respectively, to construct Bayesian inference (BI) tree and Neighbor-joining (NJ) phylogenetic tree. The results were consistent. *Punica granatum* ‘Nana’ and its original species *Punica granatum* were nested together into a monophyletic group with 100% bootstrap support, which was closely related to *Heimia myrtifolia* (Figure 1).

**Figure 1.**
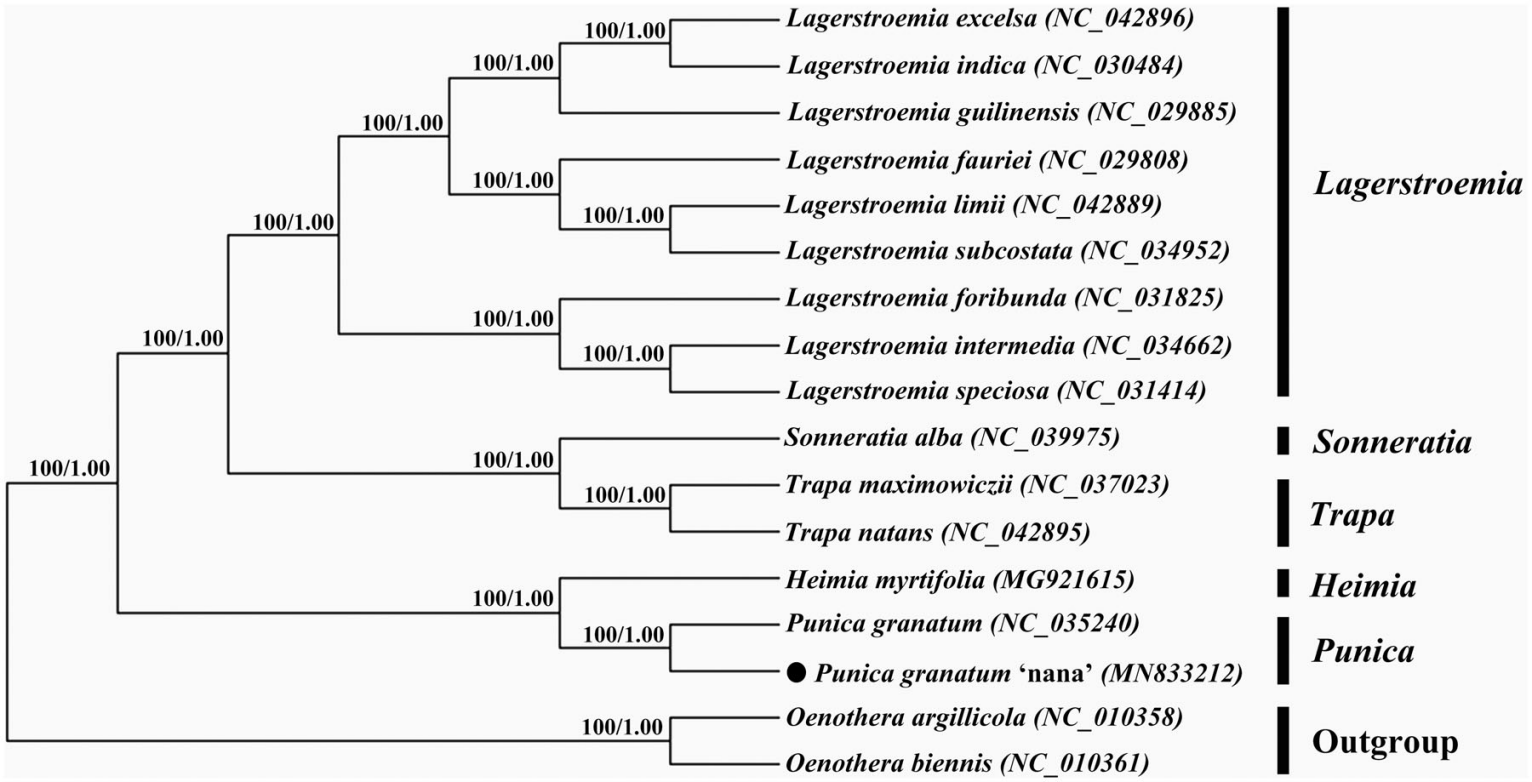
Phylogenetic tree based on chloroplast genome sequences of 15 Lythraceae species with two Onagraceae species as outgroups. Numbers above branches indicated the bootstrap value of NJ (left) and BI (right) methods.

## Data Availability

The data that support the findings of this study are openly available in GenBank at https://www.ncbi.nlm.nih.gov/genbank/, reference number: MN833212.
